# Highly Sensitive Surface Acoustic Wave Sensors for Ammonia Gas Detection at Room Temperature Using Gold Nanoparticles–Cuprous Oxide/Reduced Graphene Oxide/Polypyrrole Hybrid Nanocomposite Film

**DOI:** 10.3390/polym17081024

**Published:** 2025-04-10

**Authors:** Chung-Long Pan, Tien-Tsan Hung, Chi-Yen Shen, Pin-Hong Chen, Chi-Ming Tai

**Affiliations:** 1 Department of Electrical Engineering, I-Shou University, Kaohsiung 84001, Taiwan; ptl@isu.edu.tw (C.-L.P.); isu11102005m@cloud.isu.edu.tw (P.-H.C.); 2 Department of Chemical Engineering, I-Shou University, Kaohsiung 84001, Taiwan; 3 Division of Gastroenterology and Hepatology, Department of Internal Medicine, E-Da Hospital, I-Shou University, Kaohsiung 84001, Taiwan; ed102166@edah.org.tw

**Keywords:** surface acoustic wave, NH_3_, AuNPs-Cu_2_O/rGO/PPy, selectivity

## Abstract

Gold nanoparticles–cuprous oxide/reduced graphene oxide/polypyrrole (AuNPs-Cu_2_O/rGO/PPy) hybrid nanocomposites were synthesized for surface acoustic wave (SAW) sensors, achieving high sensitivity (2 Hz/ppb), selectivity, and fast response (~2 min) at room temperature. The films, deposited via spin-coating, were characterized by SEM, EDS, and XRD, revealing a rough, wrinkled morphology beneficial for gas adsorption. The sensor showed significant frequency shifts to NH_3_, enhanced by AuNPs, Cu_2_O, rGO, and PPy. It had a 6.4-fold stronger response to NH_3_ compared to CO_2_, H_2_, and CO, confirming excellent selectivity. The linear detection range was 12–1000 ppb, with a limit of detection (LOD) of 8 ppb. Humidity affected performance, causing negative frequency shifts, and sensitivity declined after 30 days due to resistivity changes. Despite this, the sensor demonstrated excellent NH_3_ selectivity and stability across multiple cycles. In simulated breath tests, it distinguished between healthy and patient-like samples, highlighting its potential as a reliable, non-invasive diagnostic tool.

## 1. Introduction

Gas sensors possess a broad spectrum of applications, including the detection of toxic and flammable gases, monitoring emissions from vehicles and combustion processes, conducting respiratory analyses for medical diagnostics, and ensuring quality control within the chemical, food, and cosmetic industries. In recent years, the increasing demand for environmental safety and healthcare has led to heightened attention on rapid, accurate, and highly sensitive smart gas sensors [1,2].

Within the medical domain, gas sensors serve as critical tools for early diagnosis and intervention by analyzing biomarkers in breath or other bodily fluids. For example, gas sensors can effectively monitor the progression of chronic diseases such as asthma and chronic obstructive pulmonary disease (COPD) by measuring levels of nitric oxide and other pertinent gases in exhaled breath. This allows healthcare practitioners to make timely adjustments to treatment plans [3,4,5]. Furthermore, the analysis of gas composition in breath can facilitate the diagnosis of various diseases. For instance, the ammonia (NH_3_) breath test (ABT) has gained recognition as a cost-effective and non-invasive method for diagnosing *Helicobacter pylori* (*H. pylori*) infection. This test utilizes the urease enzyme produced by *H. pylori* to convert urea into NH_3_ and carbon dioxide, enabling diagnosis through the assessment of NH_3_ concentration changes in the breath. Research has demonstrated that the sensitivity and specificity of the ABT align closely with those of the rapid urease test (RUT) and histopathological examination, suggesting that NH_3_ breath testing is an inexpensive and feasible alternative for early diagnosis of *H. pylori* infection [6,7].

Gas sensors, essential across numerous applications, encompass several principal types, each distinguished by their mechanism and suitability for specific uses. Electrochemical sensors operate by inducing chemical reactions between gas molecules and an electrolyte at an electrode. These sensors exhibit remarkable sensitivity to toxic gases such as carbon monoxide, NH_3_, and hydrogen sulfide, and their low power consumption renders them particularly suitable for portable devices. Optical sensors, on the other hand, determine gas concentrations by analyzing variations in the optical properties of gas molecules. Renowned for their precision and rapid response times, these sensors are especially valuable in applications such as breath gas analysis [4]. Metal oxide semiconductor (MOS) sensors rely on chemical interactions between gas molecules and a metal oxide surface, resulting in measurable changes in electrical resistance. Valued for their high sensitivity, affordability, and simplicity, these sensors are commonly used to detect volatile organic compounds and reducing gases [8]. Meanwhile, nano-carbon-based sensors, including those utilizing graphene and carbon nanotubes, have garnered significant interest in gas detection. Boasting extraordinary sensitivity and superior performance at room temperature, these materials are ideal for identifying gases even at low concentrations. Surface acoustic wave (SAW) sensors operate on the principle of the piezoelectric effect for gas detection. When gas molecules adsorb onto or desorb from the sensing film on the SAW device, they induce changes in the electrical signal parameters, including amplitude, phase, frequency, or time delay. These signal variations can be utilized for the detection and quantification of specific gaseous analytes [9].

In the field of NH_3_ detection, composite materials are gaining increasing prominence due to their ability to synergize the strengths of multiple constituents while mitigating the limitations of individual components. By integrating materials with strong NH_3_ adsorption properties—such as metal oxides or carbon-based nanomaterials—with substrates that provide structural stability and enhanced conductivity, such as conductive polymers, researchers have developed advanced sensing materials that demonstrate exceptional sensitivity to trace concentrations of NH_3_. This approach also effectively reduces cross-sensitivity to other gases, ensuring accurate and reliable detection. For instance, composites like Cu-MOF/graphene/polyaniline and ZIF-67/rGO, which feature high surface areas and porous structures, significantly enhance both the sensitivity and selectivity of NH_3_ sensors [10,11]. Additionally, the incorporation of graphene, polyaniline, and carbon nanotubes improves the electrical conductivity of these composites, thereby accelerating their response speed and increasing their sensitivity to NH_3_ [10,11,12]. Furthermore, materials such as MoS_2_/SnO_2_ and MoS_2_/MWCNT composites have demonstrated rapid response and recovery times, which are critical for real-time NH_3_ monitoring applications [12,13]. Notably, ternary composites like SnO_2_-ZnO-Fe_2_O_3_ have shown remarkable performance, with detection limits as low as 1 ppb, coupled with excellent stability, selectivity, and specificity for both NH_3_ and ethanol detection [14]. These advancements underscore the immense potential of composite materials in developing high-performance NH_3_ sensors tailored for diverse environmental and industrial applications.

Cu_2_O, a p-type semiconductor with a direct band gap of 1.9–2.2 eV, exhibits excellent gas sensing capabilities due to its structural features. Morphologies such as cubic, hollow, rosette-shaped (composed of nanosheets), and hollow polyhedral structures enhance its specific surface area, facilitating efficient gas adsorption and reactions. These characteristics improve sensitivity and shorten response times, particularly for reducing gases like NH_3_ and H_2_S [15,16,17]. Additionally, Cu_2_O sensors operate effectively at relatively low temperatures (200–450 °C) compared to other metal oxides [18]. However, drawbacks include poor conductivity and diminished stability under certain conditions, potentially affecting long-term sensor performance [19,20]. Pure rGO demonstrates good sensitivity and fast response to NH_3_ at room temperature. Its high electrical conductivity and chemically active defect sites enable efficient gas adsorption and desorption. For instance, a chemically reduced graphene oxide sensor achieved responses of 5.5% at 200 ppm and 23% at 2800 ppm NH_3_, with rapid recovery at low concentrations without heating [21]. However, the gas sensing performance of pure rGO is limited compared to rGO–metal oxide composites. For example, a three-dimensional SiO_2_-rGO framework achieved a 31.5% response to 50 ppm NH_3_, significantly outperforming a two-dimensional rGO network’s 1.5% response [22,23]. PPy, a conductive polymer, is widely utilized in NH_3_ detection due to its high surface area, low density, porosity, good reversibility, and ability to function at room temperature [24,25]. However, pure PPy has limitations such as low selectivity, sensitivity to humidity, and relatively poor stability. Its response and recovery times are slower than those of composite materials like PPy/MoS_2_, which exhibit enhanced sensitivity and stability [26,27]. AuNPs are highly promising for NH_3_ detection owing to their exceptional catalytic activity, high specific surface area, and excellent electrical conductivity. These properties enhance the adsorption and dissociation of NH_3_ molecules, improving sensitivity, response speed, and recovery time. The size and shape of AuNPs can be customized to optimize sensing performance. Additionally, AuNPs can be combined with materials like metal oxides and graphene to further enhance selectivity and stability. Their unique optical properties also enable various detection techniques, such as electrochemical methods, surface plasmon resonance (SPR), and localized surface plasmon resonance (LSPR), offering versatile detection capabilities [28,29,30].

This study developed advanced sensing performance through the utilization of a multi-component composite material, AuNPs-Cu_2_O/rGO/PPy, as the sensing film. Each constituent contributes unique and complementary properties: AuNPs enhance the surface area and catalytic activity, Cu_2_O offers exceptional selectivity and sensitivity toward NH_3_, rGO improves electrical conductivity and provides an expansive surface area, while PPy supports room-temperature operation with excellent flexibility and mechanical stability. The synergistic interplay among these components significantly boosts the sensor’s response time, stability, and overall performance, ensuring long-term operational reliability. When integrated into a surface acoustic wave (SAW) device, this hybrid composite exhibited outstanding sensitivity for NH_3_ detection, maintaining rapid response and recovery times even at low concentrations. These characteristics highlight the composite’s immense potential for applications in fields such as medical diagnostics and environmental monitoring, where precision and reliability are paramount.

## 2. Materials and Methods

### 2.1. Preparation of AuNPs-Cu_2_O/rGo/PPy Hybrid Nanocomposite Films

The pyrrole monomer (Acros, Geel, Belgium) was acquired and purified through reduced-pressure distillation prior to use. Other reagents utilized include copper (II) chloride (CuCl_2_, SHOWA, Tokyo, Japan), citric acid trisodium salt (>98%, Sigma-Aldrich, St. Louis, MO, USA), polystyrene sulfonic acid (PSS, ALFA, Ward Hill, MA, USA), gold nanoparticles (50 nm diameter, OD 1, stabilized in citrate buffer), sodium dodecyl sulfate (SHOWA), and ammonium peroxydisulfate (APS, SHOWA), all of which were used without further purification. All the chemicals used were of analytical grade. NH_3_ gas (50 ppb and 1000 ppb) was obtained from Jing-De Gas Co. (Kaohsiung, Taiwan).

#### 2.1.1. Preparation of Cu_2_O/rGO Nanocomposites

**Figure 1:**
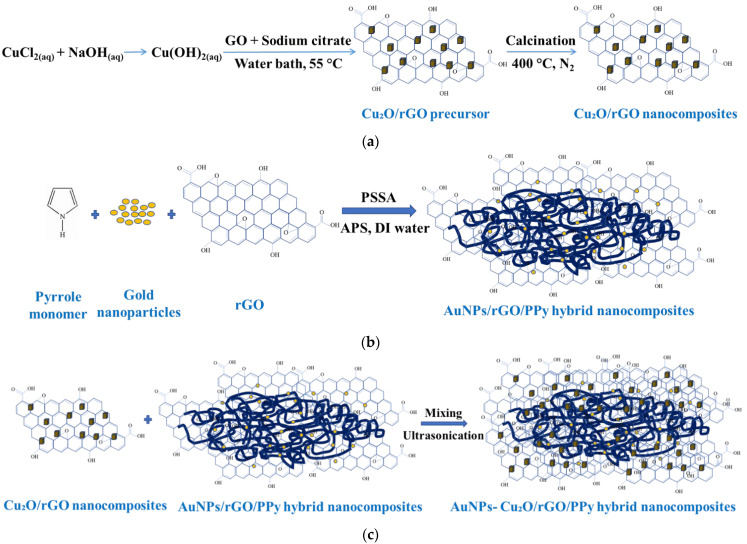
Schematic for the preparation of (**a**) Cu_2_O/rGO nanocomposites, (**b**) AuNPs/rGO/PPy hybrid nanocomposites, and (**c**) AuNPs-Cu_2_O/rGO/PPy hybrid nanocomposites.

The synthesis of GO and rGO followed a previously established method [31]. Initially, 20 mL of a 0.01 M CuCl_2_ aqueous solution was combined with 2.0 mL of a 2.0 M NaOH solution, and the mixture was continuously stirred for 30 min. Subsequently, 2.0 mg of GO and 2.0 mL of a 0.6 M trisodium citrate solution were added, facilitating the reduction of Cu(OH)_2_ to Cu_2_O and the simultaneous conversion of GO to rGO. The resulting brown solution was then heated in a water bath at 55 °C for 5 h. Afterward, the solution was rinsed several times in an ethanol–water solution (anhydrous ethanol: DI = 1:99) to remove impurities. Finally, the dried material was annealed at 400 °C for 2 h in a nitrogen atmosphere to yield the Cu_2_O/rGO nanocomposites. The entire preparation process of the Cu_2_O/rGO nanocomposites is illustrated in Figure 1a.

#### 2.1.2. Preparation of AuNPs/rGO/PPy Nanocomposites

The AuNPs/rGO/PPy nanocomposite solution was synthesized via an in situ chemical oxidative polymerization method. First, 13.3 mL of PSS was dissolved in 6.7 mL of distilled water, and a magnetic stirrer was placed into the reaction container. Then, 0.3 g of rGO, along with 0.5 g to 2.0 g of gold nanoparticle suspension, and 0.1 g of surfactant solution (SDS) were added to 10 mL of distilled water. The solution was ultrasonicated for 3 h to create a soft template. Next, 0.5 g of freshly distilled pyrrole monomer was slowly added dropwise to the stirred solution, which was kept under continuous stirring in an ice bath for 30 min. Subsequently, 2.0 g of APS was dissolved in 10 mL of distilled water and gradually introduced into the mixture. The polymerization reaction proceeded for 3 h at 5 °C with continuous stirring. The resulting AuNPs/rGO/PPy nanocomposite spin-coating solution was then ready for use. Figure 1b illustrates the preparation process.

#### 2.1.3. Preparation of AuNPs-Cu_2_O/rGO/PPy Nanocomposites

The AuNPs-Cu_2_O/rGO/PPy nanocomposites were synthesized by blending 0.05 g of the rGO/Cu_2_O composite with 20 mL of the AuNPs/rGO/PPy spin-coating solution. The mixture was thoroughly stirred to achieve a uniform dispersion and subsequently subjected to ultrasonication for 20 min to ensure homogeneous mixing. The detailed preparation process of the AuNPs-Cu_2_O/rGO/PPy hybrid nanocomposite spin-coating solution is depicted in Figure 1c.

The structural and compositional characteristics of the AuNPs-Cu_2_O/rGO/PPy hybrid nanocomposite films were investigated using various analytical techniques: Surface morphology and elemental composition were examined using an environmental scanning electron microscope (ESEM; Quanta 200, FEI, Hillsboro, OR, USA) equipped with energy-dispersive X-ray spectroscopy (EDS). Crystalline structure analysis was performed via X-ray diffraction (XRD) using a Siemens D5000 diffractometer (Bruker, Billerica, MA, USA). Electrical properties, specifically the resistivity of the hybrid nanocomposite film, were determined using a Hall effect measurement system (HMS-3000, Ecopia, Anyang, Republic of Korea).

### 2.2. SAW Sensor Fabrication

**Figure 2:**
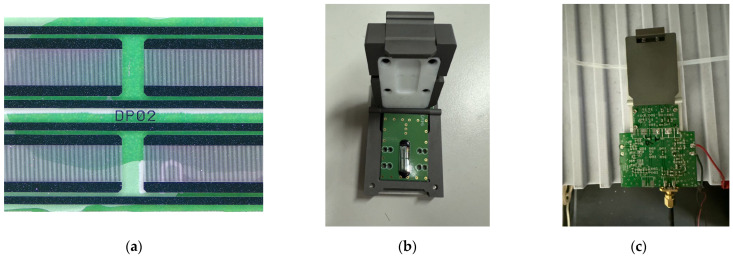
(**a**) Optical images of a dual track of interdigitated area electrodes, (**b**) Teflon sensing chamber, and (**c**) sensing system with oscillation circuit.

In this research, a dual-port surface acoustic wave (SAW) resonator was developed to operate at a frequency of 98.5 MHz on an ST-cut quartz substrate, with a design that includes stress compensation for improved NH_3_ detection. To reduce environmental interference, a dual-track setup was employed, as outlined in prior studies [32]. Each channel features input and output interdigital transducers (IDTs) utilizing an electrode-width-controlled single-phase unidirectional transducer (EWC/SPUDT) configuration. Furthermore, reflection gratings are strategically placed on either side of the channel to enhance resonance performance. The optical structure of the dual-track interdigital electrode is depicted in Figure 2. The sensing layer, made from an AuNPs-Cu_2_O/rGO/PPy solution, was applied through spin-coating onto a 1.5 × 0.5 mm^2^ area situated between the input and output IDTs, and it was subsequently annealed at 80 °C for one hour to achieve optimal performance. To achieve accurate and stable gas sensing, the surface acoustic wave (SAW) sensor was integrated into a Teflon-based sensing chamber. This design facilitates controlled gas flow and minimizes external interference. Figure 2b,c display optical images of the assembled sensing chamber and its connection to the oscillation circuit, respectively. The chamber features designated gas inlet and outlet ports that ensure proper gas flow across the sensing region. This configuration aligns the exposure of the test gas with the AuNPs-Cu_2_O/rGO/PPy sensing layer, which is located between the input and output interdigitated transducers (IDTs).

### 2.3. Gas Detection Measurement

**Figure 3:**
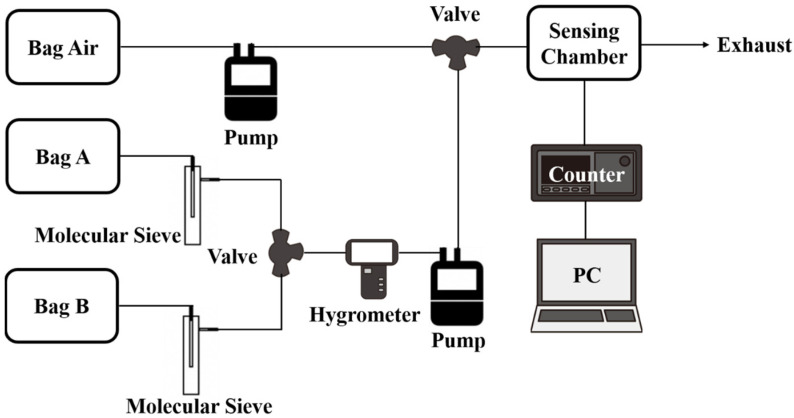
Experimental setup for NH_3_ gas sensing in human breath.

The prepared SAW sensor detected gaseous analytes by monitoring working frequency changes caused by target gas adsorption. During experiments, a frequency counter (53,132 A; Agilent Technologies, Santa Clara, CA, USA) captured frequency variations at 1 Hz intervals, instantly transmitting data to a computer for real-time analysis. This study encompassed two experimental settings: dry and humid environments. Refer to our previous works [33,34] for comprehensive experimental setup details. The sensing properties were measured in an enclosure with various NH_3_ concentrations. Mass flow controllers (MFCs) regulated the ratio of NH_3_ to dry air from certified cylinders. An NH_3_ sensor verified gas concentration, while measurements occurred in a temperature-stabilized 5 cm^3^ sensing chamber. The frequency changes of the SAW sensor were measured using a frequency counter (53,132 A, Agilent, CA, USA). Testing involved introducing dry air for 30 min to stabilize conditions, followed by the NH_3_ gas mixture for 3 min, and then dry air again for 30 min to complete one cycle. After experiments, the sensor was stored in a nitrogen-filled container to prevent contamination. To evaluate the capability of the proposed SAW sensor for human breath analysis, we conducted simulated detection experiments comparing healthy human breath and simulated patient breath. The experimental setup is illustrated in Figure 3, with ambient humidity maintained at 35% RH. Commercial gas sampling bags were used to collect human breath samples. Three bags were employed, labeled Air, A, and B. The airbag contained high-purity dry air, bag A held healthy human breath, and bag B contained simulated patient breath (including 100 ppb NH_3_). Samples from bags A and B passed through separate tubes filled with molecular sieves (Type 3A, 1–2 mm beads) to remove moisture. A hygrometer monitored ambient humidity. The experimental procedure involved sequentially pumping contents from Air, A, and B bags into the SAW sensor chamber (flow rate: 110 mL/min) for 3 min each. The frequency response induced by each sample on the SAW device was measured.

The sensor’s response was determined using the following equation:

∆ƒ/ƒ_0_ = (ƒ_s_ − ƒ_0_)/ƒ_0_, (1)

where ƒ_0_ represents the baseline frequency and fs indicates the maximum frequency reached when exposed to the target gas. The response time was defined as the duration required to reach 90% of the total frequency shift. Likewise, the recovery time was measured as the period needed for the frequency to return to 90% of its initial reading after exposure to the gas.

## 3. Results

### 3.1. Material Analysis of the AuNPs-Cu_2_O/rGO/PPy Hybrid Nanocomposite Films

**Figure 4:**
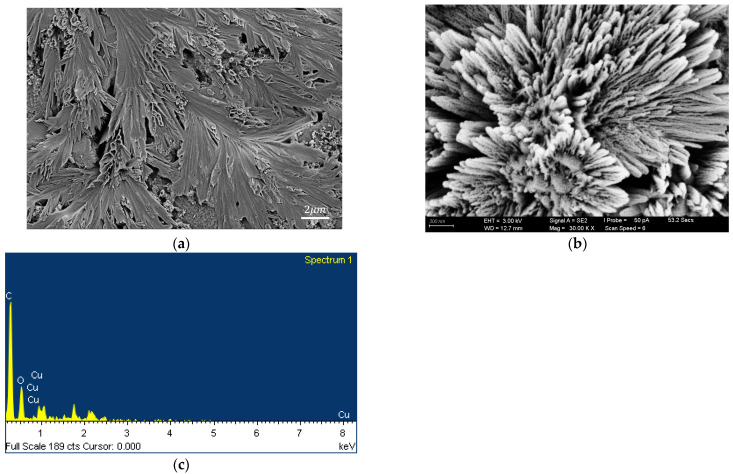
(**a**,**b**) Top-view SEM images and (**c**) EDS spectra of the Cu_2_O/rGO films.

**Figure 5:**
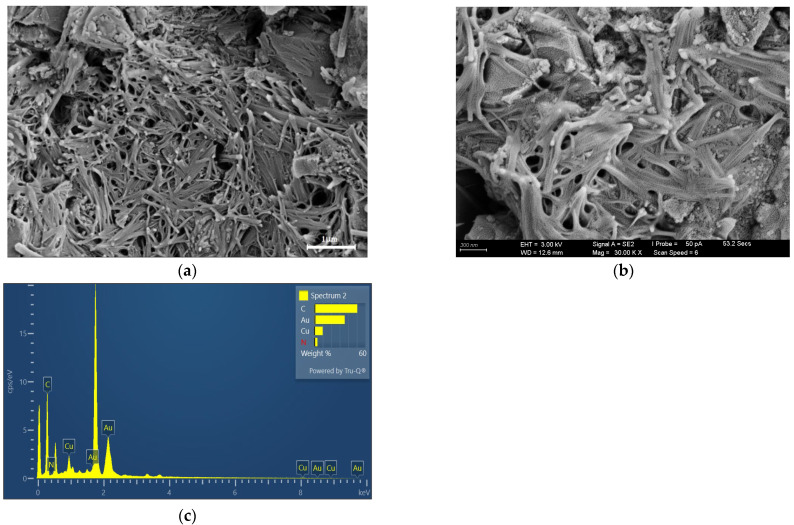
(**a**,**b**) Top-view SEM images and (**c**) EDS spectra of the AuNPs-Cu_2_O/rGO/PPy hybrid nanocomposite films.

Material analyses were performed on the Cu_2_O/rGO and AuNPs-Cu_2_O/rGO/PPy films to investigate their morphological and compositional properties. Figure 4a,b present SEM images of the Cu_2_O/rGO hybrid nanocomposite film at various magnifications, showcasing its unique flower-cluster morphology. This structure arises from the combination of Cu_2_O and rGO, with rGO sheets appearing as multilayered, stacked formations. Figure 4c displays the EDS elemental analysis, which confirms the presence of carbon (C), oxygen (O), and copper (Cu), thereby verifying the successful integration of rGO and Cu_2_O in the composite. Figure 5a,b presents an SEM image of the AuNPs-Cu_2_O/rGO/PPy hybrid nanocomposite film, where granular regions signify the distribution of AuNPs. The image reveals a highly porous surface structure interspersed with interconnected fibrous features. This morphology is attributed to the synergistic effects of the polymeric properties of PPy, the layered structure of rGO, and the embedded Cu_2_O nanoparticles, forming a porous, well-networked matrix. In Figure 5c, the EDS elemental analysis of the AuNPs-Cu_2_O/rGO/PPy film highlights the presence of C, Au, Cu, and N, corresponding to the rGO, AuNPs, Cu_2_O, and PPy, respectively, which affirms the expected composition of the hybrid nanocomposite. Both nanocomposite films exhibit intricate nanostructures and high-purity elemental compositions, featuring remarkable porosity and large surface areas. Notably, the AuNPs-Cu_2_O/rGO/PPy film, benefiting from the incorporation of AuNPs, Cu_2_O, and PPy, is anticipated to deliver enhanced electrical conductivity, stability, and suitability for advanced sensing applications.

**Figure 6:**
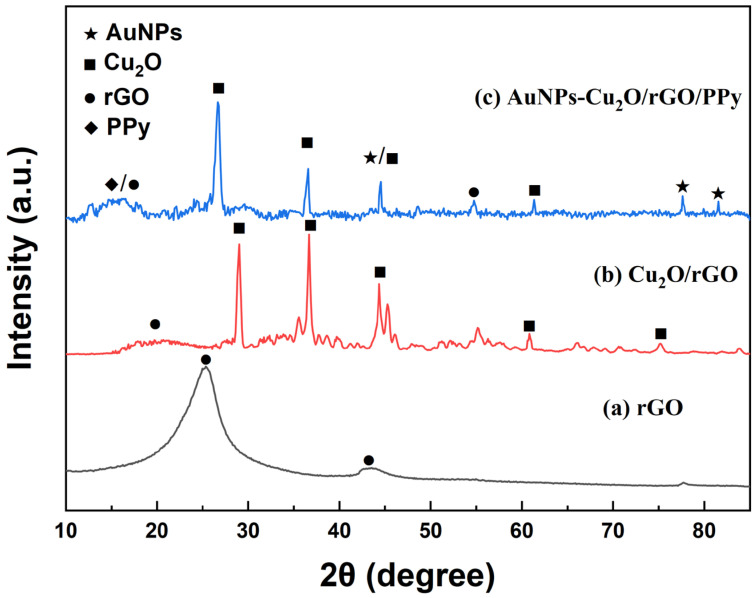
XRD patterns of (**a**) graphene, (**b**) Cu_2_O/rGO, and (**c**) AuNPs-Cu_2_O/rGO/PPy.

The XRD patterns of rGO, Cu_2_O/rGO, and AuNPs-Cu_2_O/rGO/PPy are presented in Figure 6a–c, respectively. In Figure 6a, a broad diffraction peak is observed at approximately 2θ = 25.5°, which corresponds to rGO and signifies the presence of a π-conjugated graphene structure. The broad (002) peak suggests a randomly oriented crystal phase, indicating a disrupted or loosely stacked graphene layer arrangement. Additionally, a weaker diffraction peak at 2θ = 42.60°, associated with the (100) orientation, is attributed to the turbostratic disorder of carbonaceous materials [35], further confirming the structural irregularities within rGO. In Figure 6b, in addition to the (002) peak of rGO, several characteristic diffraction peaks corresponding to cuprous oxide (Cu_2_O) are distinctly observed. These Cu_2_O peaks appear at 28.9°, 36.6°, 44.3°, 60.8°, and 75.1°, which are indexed to the (110), (111), (200), (220), and (311) crystallographic planes of Cu_2_O, respectively (JCPDS file no. 05-0667). The pronounced intensity of these peaks indicates the high crystallinity of Cu_2_O within the composite. Moreover, the broad (002) peak of rGO exhibits a noticeable shift to 20.0°, signifying an increase in the interlayer spacing (d-spacing) of rGO. This expansion is attributed to the intercalation of Cu_2_O nanoparticles, which disrupts the van der Waals interactions between adjacent graphene layers, leading to an increased interlayer distance. In Figure 6c, in addition to the (002) peak of rGO and the characteristic diffraction peaks of Cu_2_O, additional peaks corresponding to gold nanoparticles (AuNPs) are distinctly observed. The Au diffraction peaks appear at 44.3°, 77.6°, and 81.5°, which correspond to the (200), (311), and (222) planes of face-centered cubic (FCC) gold, respectively (JCPDS file no. 04-0784). Furthermore, the broad (002) peak of rGO undergoes a further shift to 16.5°, indicating a significant expansion in the interlayer spacing of rGO. This enlargement is attributed to the intercalation of both Cu_2_O and Au nanoparticles, which disrupts the stacking order of graphene layers and enhances the interlayer distance. These diffraction patterns provide compelling evidence for the successful incorporation of Cu_2_O and AuNPs within the composite matrix, corroborating the structural integrity and elemental composition of the AuNPs-Cu_2_O/rGO/PPy nanocomposite.

The resistivity of the AuNPs-Cu_2_O/rGO/PPy hybrid nanocomposite film was evaluated using a Hall effect measurement system (HMS-3000, Ecopia, Anyang, Republic of Korea). Initially, the film exhibited a resistivity of 1.468 × 10^−1^ Ω·cm. Upon exposure to 800 ppb of NH_3_ gas for 3 min, the resistivity significantly increased to 5.503 × 10^2^ Ω·cm. Subsequent desorption in dry air for 30 min restored the resistivity to 1.539 × 10^−1^ Ω·cm, indicating the film’s reversible response to NH_3_ gas. The observed increase in resistivity upon NH_3_ adsorption underscores the p-type semiconducting behavior of the AuNPs-Cu_2_O/rGO/PPy hybrid nanocomposite film. This response can be attributed to the interaction of NH_3_ molecules with the film, which withdraws charge carriers (holes) from the material, leading to a reduction in conductivity. The reversible nature of the resistivity changes highlights the potential of this nanocomposite for sensitive and reliable NH_3_ detection.

### 3.2. Gas Sensing Performance

**Figure 7:**
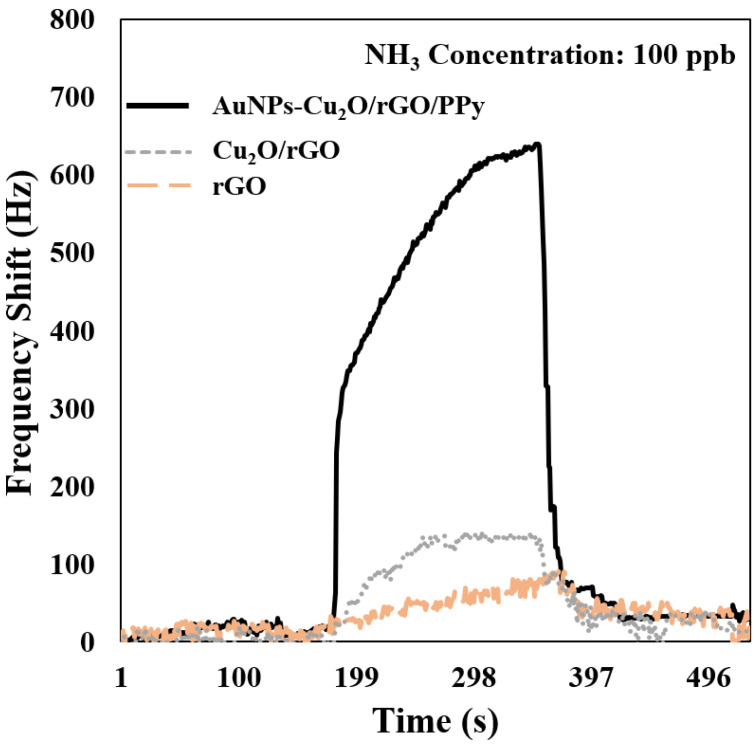
Frequency transient response of the SAW sensor with rGO, Cu_2_O/rGO, and AuNPs-Cu_2_O/rGO/PPy films to 100 ppb NH_3_ in dry air at room temperature.

Figure 7 shows the frequency transient response of SAW sensors coated with rGO, Cu_2_O/rGO, and AuNPs-Cu_2_O/rGO/PPy nanocomposite films (containing 0.5 g of gold nanoparticles) when exposed to 100 ppb NH_3_ in dry air at room temperature. Table 1 presents the frequency shift, response time, and recovery time for the three films. The layered structure of the rGO film, combined with the lack of additional functionalization, limited its capacity to effectively capture gas molecules. This limitation was reflected in its minimal frequency shift of 94 Hz, indicating low sensitivity to NH_3_. Moreover, the response and recovery times of the rGO-coated SAW sensor were relatively prolonged, underscoring the low efficiency of rGO in adsorbing and releasing NH_3_ molecules. When the SAW sensor was coated with the Cu_2_O/rGO nanocomposites, a notable improvement was observed. The frequency shift increased, and both the response and recovery times were significantly reduced. This enhancement can be attributed to the synergistic interaction between Cu_2_O and rGO, with Cu_2_O particles providing abundant active sites for NH_3_ adsorption. This combination effectively improved the sensitivity of the material and the overall performance of the sensor. The AuNPs-Cu_2_O/rGO/PPy-coated SAW sensor exhibited the most pronounced performance enhancements, achieving a frequency shift of approximately 640 Hz, which was substantially higher than that of the other two films. This remarkable sensitivity is attributed to the synergistic contributions of AuNPs and PPy. The incorporation of AuNPs provided additional active sites for NH_3_ adsorption, facilitated the generation of oxygen defects that promoted NH_3_ gas molecule reaction, and induced the spillover effect, thereby enhancing adsorption efficiency. Meanwhile, doped PPy, as a highly conductive polymer, improved the electrical conductivity of the composite, further amplifying its sensing response. Moreover, the sensor demonstrated the shortest response time (131 s) and recovery time (86 s), likely due to the combined contributions of AuNPs, Cu_2_O, and PPy. These constituents collectively enhanced the surface activity and charge transport properties of the sensing film, culminating in superior NH_3_ detection performance.

Based on Figure 7 and Table 1, it is clear that the addition of AuNPs and PPy significantly improved the frequency shift response of the sensing film to NH_3_. To further explore the effect of the amount of AuNPs added, different formulations of the AuNPs-Cu_2_O/rGO/PPy sensing film were prepared by incorporating 0.5 g, 1 g, 1.5 g, and 2 g of gold nanoparticles. However, experimental results showed that the composite film containing 0.5 g of AuNPs exhibited the best short-term stability. Therefore, the AuNPs-Cu_2_O/rGO/PPy nanocomposite film with 0.5 g of AuNPs was selected for further NH_3_ sensing characteristic studies, including linear response, sensitivity, reproducibility, response and recovery time, selectivity, long-term stability, humidity effects, and human breath analysis.

**Figure 8:**
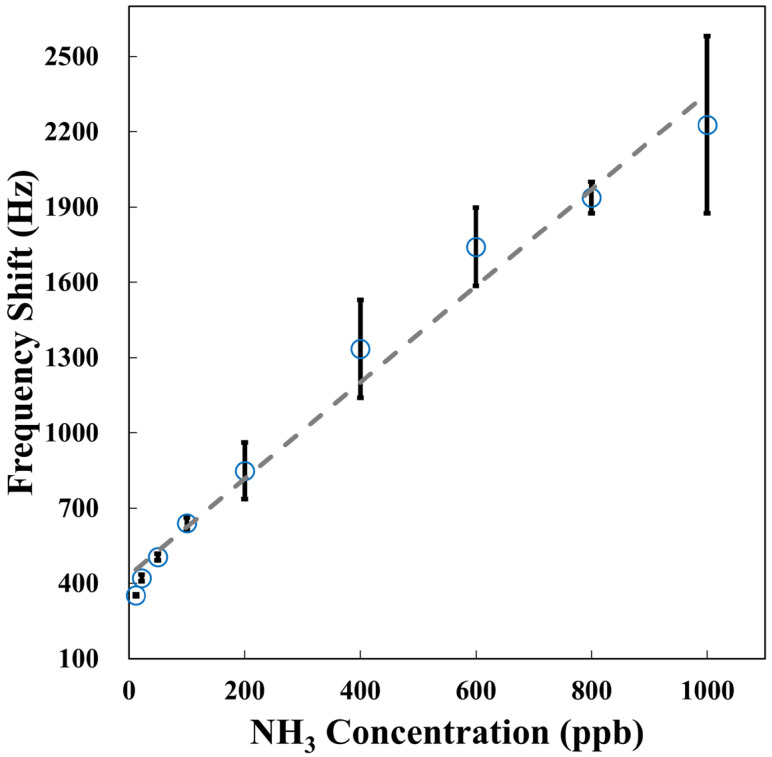
The frequency shift of the developed SAW sensor as a function of NH_3_ concentrations in dry air.

The SAW sensor, which is coated with the AuNPs-Cu_2_O/rGO/PPy nanocomposite film developed in this research, was employed to detect NH_3_ in dry air at room temperature. The results of the frequency shift are illustrated in Figure 8, with each concentration value representing the average of three experimental trials. The figure shows that the frequency shift of the SAW sensor increased linearly with increasing NH_3_ concentration in the range of 12–1000 ppb, demonstrating a linear response. The frequency response of the sensor reached saturation when detecting NH_3_ concentrations higher than 1000 ppb. Table 2 displays the frequency shifts, response times, and recovery times of the proposed SAW sensor when exposed to different levels of NH_3_ in dry air. The sensitivity (S) of the sensor refers to the change in output frequency per unit change in gas concentration, and it is calculated using the following formula: Sensitivity = ∆f/∆C, where ∆f indicates the change in frequency response, and ∆C indicates the change in NH_3_ concentration. After calculation, the sensitivity of the SAW sensor with the AuNPs-Cu_2_O/rGO/PPy nanocomposite film for NH_3_ concentrations ranging from 12 to 1000 ppb was 2 Hz/ppb, indicating sufficient sensitivity for NH_3_ detection. Furthermore, the sensor’s limit of detection (LOD) is typically defined as three times the signal-to-noise ratio (S/N). In this case, the frequency shift for detecting 12 ppb NH_3_ was 352 Hz, with the noise level measured at 75 Hz, resulting in an S/N ratio of 4.69. Thus, the estimated minimum LOD of this sensor is 8 ppb.

When SAW devices operate as sensors, gas adsorption can trigger various interactions with the sensing film, resulting in frequency shifts. For example, mechanical coupling occurs as the SAW displaces the adsorbed mass on the surface, leading to mass loading and elastic or viscoelastic effects due to the deformation of the film. Additionally, electrical coupling results in acoustoelectric interactions between the electric fields generated by the SAW and charge carriers in a conductive film. The resulting changes in wave propagation characteristics after gas adsorption can be described by the following equation:

(2) $$\frac{∆ƒ}{{ƒ}_{0}}≅\frac{∆\nu }{\nu_{0}}=-c_{m}{ƒ}_{0}∆\left(\frac{m}{A}\right)+{4c}_{e}{ƒ}_{0}\left(∆hG^{’}\right)-\frac{K^{2}}{2}∆\frac{1}{{\left(\nu_{0}C_{s}/\sigma_{s}\right)}^{2}+1},$$

where c_m_ and c_e_ are the coefficients of mass sensitivity and elasticity, respectively, (m/A) is the change in mass per unit area, h is the thickness of the sensing layer, G’ denotes the shear modulus, K^2^ is the electromechanical coupling coefficient, σ_s_ is the sheet conductivity, Cs is the capacitance per unit length of the SAW substrate, and v_0_ is the initial wave velocity of the sensor. In Equation (2), the first term accounts for the mass loading effect, which results in a negative frequency shift. The second and third terms represent the elastic properties of the sensing layer and the acoustoelectric effect, respectively, both contributing positive frequency shifts since NH_3_ acts as a reducing gas [36]. As shown in Figure 8, the SAW sensor exhibited a positive frequency shift upon detecting NH_3_, indicating that the combined effects of the acoustoelectric interactions and elastic properties outweighed the impact of mass loading.

Hall effect measurement revealed that the AuNPs-Cu_2_O/rGO/PPy nanocomposite film exhibited increased electrical resistance when exposed to NH_3_ gas. This increase led to a positive contribution in the third term (acoustoelectric effect) on the right side of Equation (2). When combined with the second term (elastic effect), these positive contributions outweighed the negative change caused by mass loading (first term). This net positive effect explains the SAW sensor’s positive frequency response observed in Figure 8.

**Figure 9:**
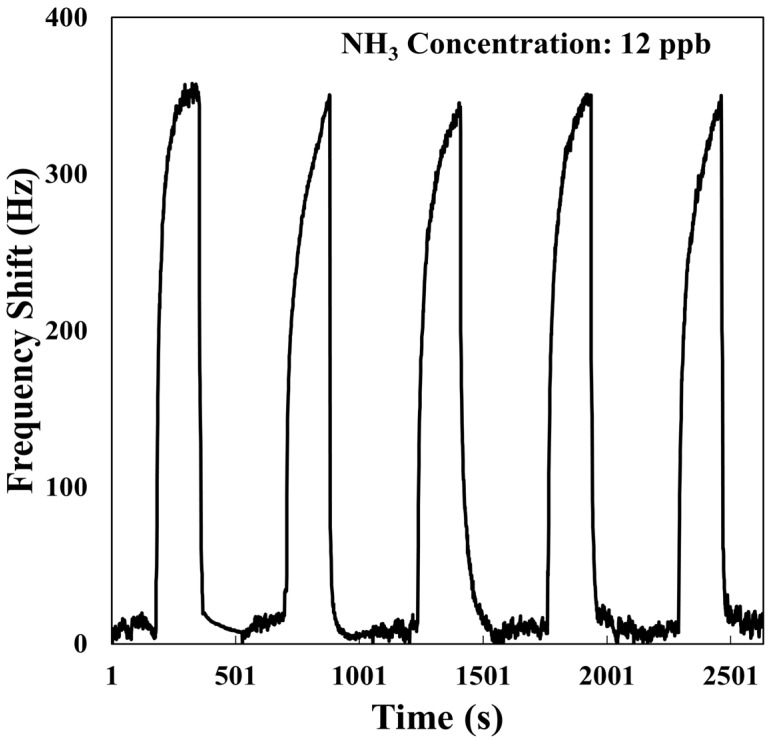
Dynamic responses of SAW sensor based on AuNPs-Cu_2_O/rGO/PPy nanocomposite film to 12 ppb NH_3_ for five consecutive cycles.

Sensors with high repeatability deliver consistent results, ensuring reliability for repeated measurements. Figure 9 illustrates the repeatability tests conducted for detecting 12 ppb of NH_3_ using the SAW sensor coated with the AuNPs-Cu_2_O/rGO/PPy hybrid nanocomposite film. The repeatability formula is Repeatability = (∆ƒ_n_/∆ƒ_1_) × 100%, where ∆f_1_ is frequency change in the first cycle, and ∆f_n_ is frequency change in the nth cycle. Across five 12 ppb NH_3_ detection cycles, the frequency shifts were 356, 350, 345, 350, and 350 Hz. The reproducibility rate was 98% over five cycles. This figure shows that the fabricated SAW sensor was able to provide consistent results during repeated sensing of 12 ppb NH_3_, demonstrating excellent repeatability.

**Figure 10:**
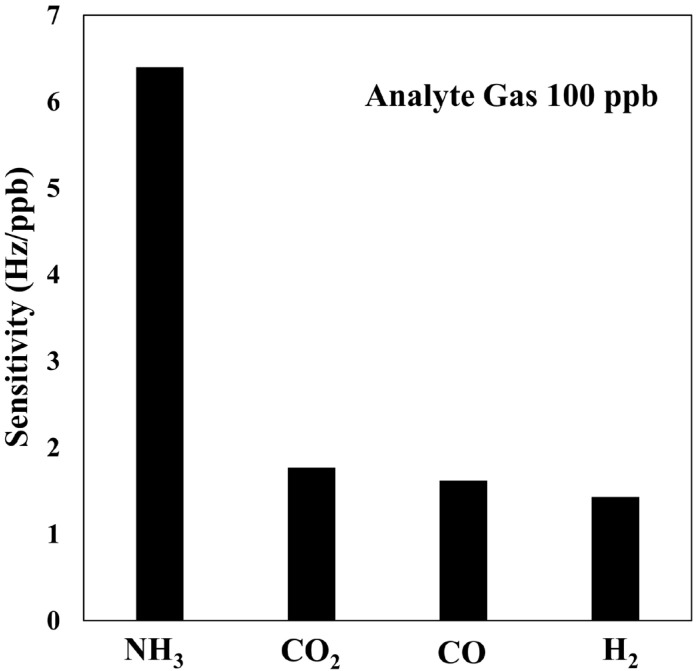
Sensitivity of a SAW sensor to 100 ppb NH_3_, CO_2_, H_2_, and CO.

A crucial characteristic of gas sensing devices is their selectivity, which denotes the capacity to accurately identify the intended analyte in the presence of potentially interfering gaseous species. As illustrated in Figure 10, the SAW sensor under investigation exhibited remarkable selectivity. Its response to NH_3_ at a concentration of 100 ppb was found to be 6.4-fold greater than its reactivity to equivalent concentrations of potential interferents, including CO_2_, H_2_, and CO.

The temporal evolution of the SAW sensor’s response to NH_3_ at 100 ppb in a dry air environment at ambient temperature is presented in Table 3. This sensor incorporates a hybrid nanocomposite film composed of AuNPs-Cu_2_O/rGO/PPy. The sensor exhibited a gradual decrease in NH_3_ sensitivity over time, as evidenced by the diminishing frequency shifts: 640 Hz initially, 424 Hz after a fortnight, and 413 Hz at the end of a month. To assess the sensor’s long-term stability, a ratio was calculated using the following formula: (∆f_Dn_/∆f_D1_) × 100%, where ∆f_D1_ and ∆f_Dn_ denote the frequency alterations on the first day and the nth day, respectively. The results show that the frequency shift of the fabricated SAW sensor, when detecting 100 ppb NH_3_ gas, maintained over 80% stability within the first three days. However, as time progressed, the frequency shift decreased to 66.3% of the initial value after 15 days and further dropped to 64.5% by the 30th day.

To investigate the decline in sensor sensitivity, the resistivity of the AuNPs-Cu_2_O/rGO/PPy hybrid nanocomposite film was measured using a Hall measurement analyzer. On the 8th day after coating, the resistivity was recorded as 7.859 × 10^−1^ Ω/cm, representing a significant increase compared to the 1.468 × 10^−1^ Ω/cm measured on the 1st day. This rise suggests a gradual degradation in the conductivity of the doped PPy over time. The reduced conductivity of doped PPy is likely attributed to its dedoping under ambient conditions, resulting in a corresponding increase in the film’s resistivity. This degradation adversely impacted the overall conductivity of the sensing film, leading to a decline in sensor sensitivity. These findings underscore the pivotal role of doped PPy in preserving the stability and performance of the nanocomposite film.

**Figure 11:**
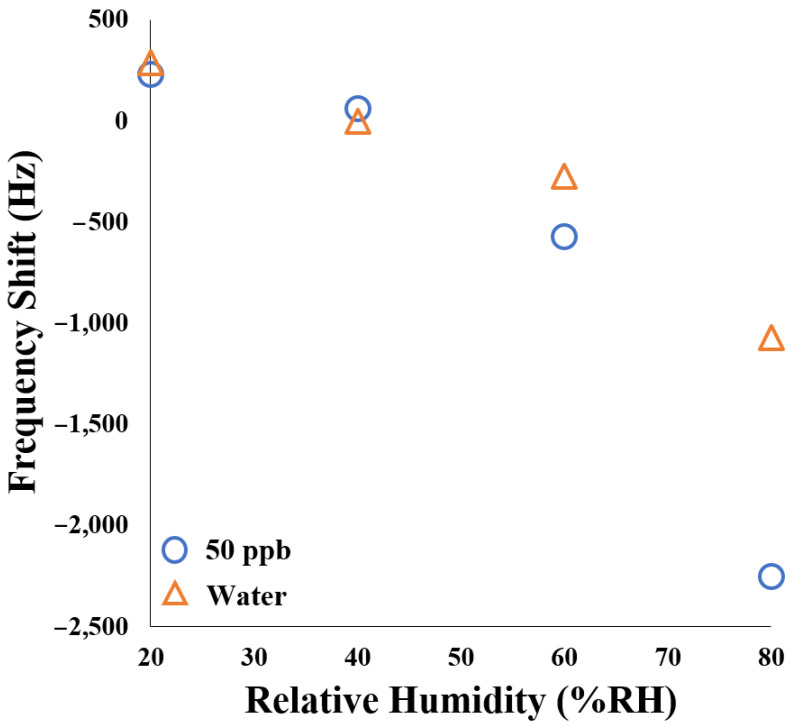
The frequency shift of the AuNPs-Cu_2_O/rGO/PPy-coated SAW sensor to 50 ppb NH_3_ under humid conditions.

Figure 11 illustrates the SAW sensor’s frequency response under varying humidity conditions at room temperature. In a water vapor environment, as relative humidity increases, the frequency shift transitions from positive to negative, indicating enhanced mass loading due to water adsorption. Specifically, under high-humidity conditions (60–70% RH), the frequency shift reached approximately −1070 Hz. At relative humidity levels of 20% and 40% RH under room-temperature conditions, the frequency shifts induced by water vapor and 50 ppb NH_3_ are comparable in magnitude. When the ambient humidity exceeds 40% RH, a distinctive pattern emerges: the negative frequency shift caused by 50 ppb NH_3_ progressively surpasses the frequency change induced by water vapor as humidity increases. This phenomenon can be attributed to the strong affinity between NH_3_ and H_2_O molecules [37]. The presence of atmospheric water molecules facilitates enhanced NH_3_ capture by the AuNPs-Cu_2_O/rGO/PPy nanocomposite film. Consequently, this leads to increased mass loading, which becomes more pronounced with rising humidity levels.

**Figure 12:**
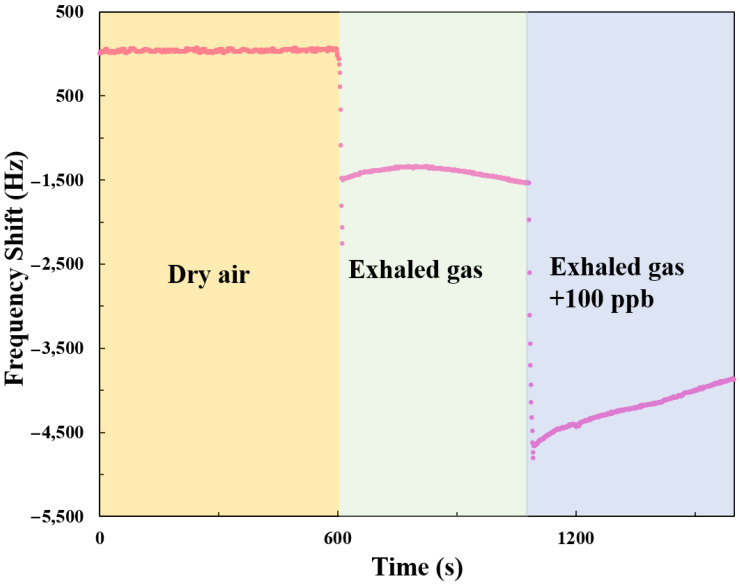
Dynamic frequency response of a SAW sensor with AuNPs-Cu_2_O/rGO/PPy nanocomposite films detecting dry air, healthy human breath, and simulated patient breath (containing 100 ppb NH_3_).

In simulated detection experiments comparing healthy human breath and simulated patient breath, the dynamic response characteristics of the SAW sensor were investigated under three distinct conditions: dry air, healthy human breath, and simulated patient breath (containing 100 ppb NH_3_), as demonstrated in Figure 12. When the sensor was exposed to dry air, the frequency response remained stable with no significant variation, indicating that the sensor has stability in the absence of NH_3_ and water vapor interference. When healthy human breath was introduced into the system, a negative frequency shift occurred (approximately −1500 Hz). This is likely due to the low concentration of NH_3_ in healthy human breath, and the presence of water vapor increases the mass loading on the sensor, leading to a negative frequency change. Furthermore, when simulated patient breath containing 100 ppb NH_3_ was introduced, the frequency dropped significantly (around −4500 Hz). This indicates that the sensor is highly sensitive to the NH_3_ concentration (100 ppb) in the simulated patient breath. This is consistent with the range of NH_3_ concentration in the breath of patients infected with *H. pylori*, suggesting that the proposed SAW sensor shows promising potential for disease diagnosis through breath analysis.

Table 4 shows some of the NH_3_ sensors operating at room temperature reported in the literature [38,39,40,41,42,43]. Based on the data presented in Table 4, the present EWC/SPUDT SAW sensor coated with a AuNPs-Cu_2_O/rGO/PPy hybrid nanocomposite film demonstrates competitive performance compared to other reported sensors in the literature. The proposed SAW sensor exhibits significant sensitivity to NH_3_ detection. The apparent frequency response in the present work is rapid towards ppb-level NH_3_. Hence, the present EWC/SPUDT SAW sensor demonstrates a balanced performance with good detection limits and recovery characteristics, making it a promising candidate for practical NH_3_ sensing applications.

The hybrid nanocomposite film, made of AuNPs, Cu_2_O, rGO, and PPy, demonstrates p-type semiconductor properties and efficient charge transport due to rGO’s high carrier mobility. rGO’s large surface area and unique structure also enhance NH_3_ molecule adsorption, improving the sensor’s performance, as supported by previous studies [44,45]. In dry air, oxygen molecules spontaneously adsorb on the AuNPs/rGO, Cu_2_O/rGO, and PPy surface, capturing electrons from the conduction band of the rGO/Cu_2_O nanocomposite and forming reactive electrophilic oxygen ions, O_2_^−^, as shown in the following equation:

O_2(gas)_→O_2(ads)_, (3)

(4) $$O_{2(\mathrm{ads})}+e^{-}\to {O_{2}^{-}}_{\left(\mathrm{ads}\right)}$$

When the AuNPs-Cu_2_O/rGO/PPy hybrid nanocomposite film encounters NH_3_, it triggers two concurrent mechanisms. One of these processes involves the interaction between NH_3_ molecules and O_2_^−^, resulting in the liberation of a substantial quantity of electrons. This reaction can be represented by the following chemical equation, as documented in previous research [46]:

NH_3(gas)_ → NH_3(ads)_, (5)

(6) $${4NH}_{3\left(ads\right)}+{5O}_{2\left(ads\right)}^{-}\to 4NO+{6H}_{2}O+5e^{-},$$

*h*^∙^+ *e*^−^→ null, (7)

The release of electrons onto the hybrid nanocomposite film surface during exposure to NH_3_ results in a reduction in conductivity and an increase in sensor resistance. These released electrons recombine with the holes present in the nanocomposite, leading to their neutralization. Simultaneously, an oxidation–reduction reaction occurs within the polymeric entanglement and the porous structure of PPy, facilitating the interaction with NH_3_ molecules. The sensing behavior of PPy toward NH_3_ can be attributed to the protonation/deprotonation mechanism. During NH_3_ adsorption, the deprotonation of PPy occurs as NH_3_ molecules interact with protons on the composite film surface. Conversely, during desorption, protonation restores the film’s original state. This dynamic process underpins the reversible nature of NH_3_ sensing by the nanocomposite material, as described in prior studies [47]. The reaction of PPy with NH_3_ is represented by the following equations:

Adsorption: PPy^+^ + NH_3_ → PPy^0^ + NH_4_^+^, (8)

Desorption: PPy^0^ + NH_4_^+^ → PPy^+^ + NH_3_ (9)

The interaction between NH_3_ molecules and PPy involves the transfer of electrons from NH_3_ to PPy, leading to the formation of ammonium ions and an increase in the electrical resistance of PPy. This process is reversible; the conductivity of the AuNPs-Cu_2_O/rGO/PPy hybrid nanocomposite film can be restored upon the removal of NH_3_ and exposure to air. Previous research indicates that π-π stacking may occur between the reduced rGO and the PPy layers, facilitating electron transfer between the layers [48]. Electrons generated from the NH_3_-O_2_ reaction enter the PPy layer, catalyzing a redox reaction between PPy and NH_3_. This interaction results in the deprotonation of PPy and a reduction in the number of charge carriers along its main chain. The electrons then rapidly migrate through the hybrid nanocomposite film, recombining with holes in the p-type semiconductor. As a result, the concurrent electron–hole recombination in both PPy and rGO leads to a decrease in hole density and an increase in resistance, particularly at higher NH_3_ concentrations. During the recovery phase, the hybrid nanocomposite film quickly releases electrons through reactions with adsorbed oxygen molecules, facilitating a rapid return to baseline conditions. Additionally, the incorporation of AuNPs enhances the adsorption of NH_3_ molecules on the rGO surface due to strong binding interactions. This, in turn, increases rGO’s capacity for NH_3_ adsorption, thereby improving the overall sensitivity of NH_3_ detection [49].

Thus, this study leverages the synergistic effect between rGO and PPy, combining the properties of both materials: the porous PPy layer plays an important role in electron exchange during NH_3_ sensing, and rGO, in the AuNPs-Cu_2_O/rGO/PPy nanocomposite film, not only serves as a support for Cu_2_O and PPy but also provides additional pathways for electron transfer, allowing rapid electron transport between the Cu_2_O and PPy layers. The incorporation of AuNPs not only increases the specific surface area of the sensing film but also allows NH_3_ molecules to bind more strongly to the surface of the AuNPs-Cu_2_O/rGO/PPy composite. NH_3_ gas molecules can bind more firmly to the surface of AuNPs-Cu_2_O/rGO/PPy, allowing more NH_3_ molecules to be adsorbed onto the film surface, indirectly enhancing the adsorption capacity of AuNPs-Cu_2_O/rGO/PPy for NH_3_ molecules and thereby improving the sensitivity of NH_3_ sensing. The above explanation confirms that the resistance of the AuNPs-Cu_2_O/rGO/PPy nanocomposite film increases during NH_3_ detection, leading to a positive acoustoelectric effect in the third term of Equation (2). When combined with the positive elastic effect in the second term, it exceeds the negative change in mass loading from the first term, resulting in a positive frequency response of the SAW sensor, as evidenced by Figure 8.

## 4. Conclusions

This study investigates a high-sensitivity SAW sensor for ammonia detection, utilizing AuNPs-Cu_2_O/rGO/PPy hybrid nanocomposite films as the sensing material, designed for ppb-level NH_3_ detection at room temperature. SEM, EDS, and XRD characterizations showed that the AuNPs-Cu_2_O/rGO/PPy hybrid nanocomposite films were successfully synthesized and exhibited numerous wrinkles and a rough structure, which are crucial for gas adsorption. In dry air at room temperature, the SAW sensor coated with AuNPs-Cu_2_O/rGO/PPy nanocomposite films exhibited a linear frequency shift in the 12–1000 ppb NH_3_ concentration range, with a sensitivity of 2 Hz/ppb. The sensor demonstrated a LOD of 8 ppb (S/N = 3), and at 1000 ppb, the response began to saturate, indicating the upper adsorption limit. The average repeatability for detecting 12 ppb NH_3_ was 98%, and when detecting 100 ppb interfering gases, the sensor showed 6.4 times greater selectivity, demonstrating high specificity for NH_3_. The response and recovery times for detecting 12–1000 ppb NH_3_ in dry air were both around 2 min. The sensor’s long-term stability needs improvement, as its frequency response decreased to 66.3% of the initial value by day 15. Due to NH_3_’s strong affinity for H_2_O, environmental moisture helps the AuNPs-Cu_2_O/rGO/PPy nanocomposite films capture more NH_3_ molecules, causing the frequency shift to increase as humidity rises. To assess the sensor’s feasibility for real-world applications, simulated human breath tests were conducted. The results showed that the SAW sensor could clearly distinguish between dry air, healthy human breath, and simulated patient breath (containing 100 ppb NH_3_), indicating its potential for detecting trace NH_3_ variations in human breath, which could have significant medical diagnostic value. The AuNPs-Cu_2_O/rGO/PPy nanocomposite SAW sensor demonstrated excellent sensitivity, selectivity, and fast response/recovery times for ppb-level NH_3_ detection at room temperature. Although its long-term stability needs further improvement, the sensor shows great potential for detecting NH_3_ in human breath, offering a new possibility for non-invasive diagnosis of Helicobacter pylori infections.

## Figures and Tables

**Table 1:** Sensing performance of SAW sensors coated with rGO, Cu_2_O/rGO, and AuNPs-Cu_2_O/rGO/PPy films when detecting 100 ppb NH_3_ gas in dry air.

Sample	Frequency Shift (Hz)	Response Time (s)	Recovery Time (s)
rGO	94	152	132
Cu_2_O/rGO	139	144	120
AuNPs-Cu_2_O/rGO/PPy	640	131	86

**Table 2:** Sensing performance of SAW sensor based on AuNPs-Cu_2_O/rGO/PPy hybrid nanocomposite film toward NH_3_ gas in dry air.

Concentration (ppb)	12	22	50	100	200	400	600	800	1000
Frequency shift (Hz)	352	421	505	640	848	1334	1741	1937	2227
Response time (s)	130	134	132	131	96	69	92	93	88
Recovery time (s)	96	130	87	86	72	53	66	91	85

**Table 3:** Responses of a SAW sensor coated with AuNPs-Cu_2_O/rGO/PPy sensing film to 100 ppb of NH3 gas for 30 days.

Time (Day)	1	2	3	8	15	30
Frequency shift (Hz)	640	590	539	450	424	413
Stability (%)	100.0%	92.2%	84.2%	70.3%	66.3%	64.5%

**Table 4:** Comparison of the performances of the AuNPs-Cu_2_O/rGO/PPy-based sensor developed in this work and the other NH_3_ sensors reported in the literature.

Sensing Film	Sensitivity	LOD	Response Time	Recovery Time	Reference
AlO(OH)	1540 Hz to 10 ppm	2 ppm	30–60 s	60–90 s	[38]
GO-SnO_2_	0.0098 mV/ppb	40 ppb	16 s	195 s	[39]
SnO_2_/Co_3_O_4_	3.33 Hz/ppm	9 ppm	100–120 s	30–50 s	[40]
PAni-WO_3_	121% to 100 ppm	1 ppm	32 s	388 s	[41]
Polyacrylic Acid	750 Hz/ppm	0.5 ppm	200 s	230 s	[42]
PANI–rGO	13% to15 ppm	0.3 ppm	96 s	22.1 min	[43]
AuNPs-Cu_2_O/rGO/PPy	2 Hz/ppb	8 ppb	130 s	96 s	This work

## Data Availability

The original contributions presented in this study are included in the article. Further inquiries can be directed to the corresponding authors.
